# Soft electronic skin for self-deployable tape-spring hinges

**DOI:** 10.1038/s44172-024-00163-x

**Published:** 2024-01-25

**Authors:** Yao Yao, Xin Ning

**Affiliations:** Department of Aerospace Engineering, University of Illinois at Urbana-Champaign, Urbana, IL 61801 USA

**Keywords:** Aerospace engineering, Mechanical engineering

## Abstract

Thin-walled structures utilizing the release of stored strain energy for self-deployment have gained popularity in deployable space structures. However, the integration of traditional rigid or bulky sensors to monitor their mechanical behavior presents challenges due to large local deformations and strains involved in folding and deployment. Here we introduce a concept of structural electronic skin (e-skin) that is soft, lightweight, and designed for sensing the folding and deployment of tape-spring hinges. The e-skin demonstrates the capability to accommodate significant hinge deformation and enables multimodal sensing, including strain measurements, motion sensing, and dynamics monitoring. The research showcases a promising approach that leverages the design and manufacturing of soft electronics to fulfill the requirements of thin, lightweight, and soft functional devices for multifunctionality in deployable space structures.

## Abstract (web summary)

Yao and Ning present a method for sensing the folding and deployment behavior of tape spring hinges using skin-like strain and motion sensors. The integration of these soft sensors does not affect the structural deformations, thereby providing a solution to monitor the mechanical behavior of high-strain components under large deformations.

## Introduction

We introduce an approach utilizing a soft electronic skin (e-skin) attached on self-deployable tape-spring hinges for the purpose of in-situ monitoring folding and deployment behavior. Deployable structures play a pivotal role in space exploration systems as they can be folded during launch and subsequently expanded to substantial sizes on orbit. Recent studies have proposed the utilization of elastic thin-walled booms and springs as mechanical hinges for deployable space structures^1–3^. These hinges can store elastic strain energy while in a folded state and can autonomously deploy by releasing the stored strain energy. This characteristic offers a straightforward and lightweight solution for deploying space structures, eliminating the necessity for motors or other active materials^4–6^. The folding and deployment processes of these hinges entail significant mechanical deformation and intricate dynamics, underscoring the importance of monitoring and understanding their behavior to facilitate the design and operation of such hinges.

Despite recent progress in techniques aimed at sensing the folding and deployment behavior of thin-walled deployable space structures, notable challenges still persist. Jorgensen et al. employed a charge-coupled device (CCD) camera to develop a videometry system for measuring the deployment of a solar array with z-fold hinges^7^. Laser Doppler vibrometry is another widely used method for structural health monitoring^8^. Yu et al. showcased the potential of single-camera digital image correlation (DIC) in measuring plate vibrations induced by an impulse hammer^9^. Deflectometry, a subset of DIC, holds promise as a method for measuring plate vibrations without the requirement of a speckle pattern on the test sample surface, relying solely on a reflective surface^10^. However, these optical approaches are typically limited to small deviations from the camera’s focal plane, and larger motions can lead to incorrect or inconclusive analysis. Moreover, the quality of the speckle pattern and lighting conditions significantly impact the results of DIC-based methods. For example, it was demonstrated in the study of the Roll-Out Solar Array that, due to the constantly shifting lighting conditions in space, most of the photogrammetry targets were proven to be too hard to resolve from the images taken from the International Space Station^11^. Although with the help of retroreflective silver circles, data was successfully extracted, the concerns about the adhesive affecting the deployment of the solar array resulted in avoidance of the usage of the retroreflective silver circles on many parts of the solar array^11^.

To address the limitations associated with optical-based methods, researchers have explored alternative approaches. The usage of metal-based strain sensors has been dominant in strain measuring. Despite the stability of its performance, it usually leads to a low gauge factor of approximately 2, making it difficult to sense small strain variations. Li et al. developed a diaphragm-type fiber Bragg grating (FBG) sensor^12^, which can be integrated into metal or composite materials for strain sensing. However, the integration of optical fibers into structures may introduce defects and compromise structural integrity. The inherent brittleness of FBG sensors also poses challenges for integration with highly deformable thin-walled deployable structures. Additionally, the strict requirements regarding weight and size of sensors for in-space applications further compound the challenges faced in this domain.

Recent advances in soft electronics have demonstrated the successful integration of advanced electronics in thin, lightweight, and flexible forms that can withstand substantial mechanical strains, akin to the deformations experienced by human skin^13,14^. While there have been noteworthy advancements in soft electronics, the current research has primarily concentrated on biomedical applications. Nevertheless, the favorable characteristics of soft electronics position them as promising candidates for integration with highly deformable thin-walled space structures, which necessitate thin, lightweight, and flexible functional devices for monitoring structural behavior. However, the exploration of the full potential of soft electronics in the field of space structures is still in its early stages and requires further research and investigation.

In this study, we present a concept of soft e-skin for deployable thin-walled structures, which comprises soft and stretchable circuits, highly sensitive thin-film strain sensors, and miniaturized motion sensors. These components are integrated to form a thin and lightweight functional material that can be applied to tape-spring hinges. The e-skin exhibits the capability to measure various structural characteristics, including vibration frequency, deployment and folding status, strains, and movements of folded hinge regions. This innovative approach leverages soft electronics to enable in-situ monitoring of highly deformable thin-walled space structures, showcasing the potential of soft electronics in creating multifunctional and self-sensing space structures. This study serves as a foundational work that may pave the way for future research and advancements in the application of soft electronics in space engineering.

## Results and discussion

### Soft electronic skin

**Fig. 1:**
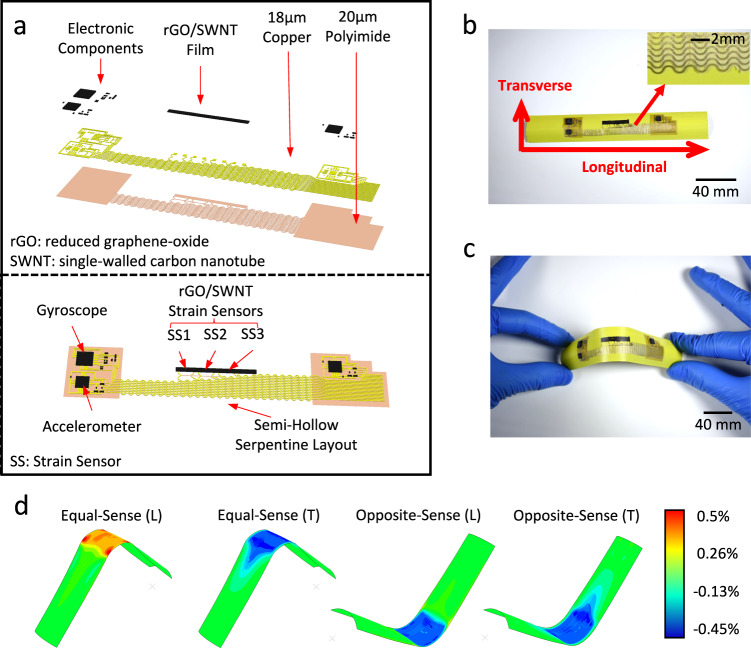
Schematic and optical images of soft e-skin and finite element analysis (FEA) simulations. **a** Schematic of exploded and integrated circuit design. **b** A section of an undeformed tape-spring hinge with the e-skin. **c** A section of the deformed tape-spring hinge with the e-skin. **d** FEA on the tape-spring hinge with attached e-skin under equal-sense and opposite-sense bending, where the color map shows the induced strain in the longitudinal direction (L) and transverse direction (T).

Figure 1a illustrates a schematic representation of the e-skin along with its exploded view. The e-skin consists of three layers. The first layer comprises a flexible polyimide (PI) film, which serves as a substrate for supporting the e-skin. The second layer consists of a pre-laminated copper (Cu) film (Dupont, AR1820) that is patterned to establish the electrical circuitry. This circuitry facilitates the interconnection of all electronic components and thin-film strain sensors, forming the third layer.

Figure 1b presents a tape-spring hinge with the e-skin bonded to its surface using a sprayed adhesive (3M^TM^ Super 77^TM^ Multipurpose Spray Adhesive). The tape-spring hinge is a 6-inch segment of a steel tape measure (Stanley 25 ft Magnetic LeverLock Tape Measure) with a thickness of 0.115 mm and a curved cross-section (with a flattened width of 25.4 mm, and a curvature of 16.9 mm). These commercially available steel tape measures have been widely employed as elastic hinges and deployable structures in spacecraft, particularly for small satellites such as CubeSats. When folded, due to the bending moment applied to the end of the tape measure, the curvature of the tape measure in the transverse direction will be reduced to zero, whereas the curvature in the longitudinal direction will increase from zero to a state to counteract the bending moment. The change in the curvature will then lead to a development of strain in the hinge area. Additionally, strains developed beyond the folded region are negligible. Such behavior has been extensively studied by researchers, providing numeric and analytic solutions^3^. Figure 1c shows an example image of a tape measure enduring an equal sense bending with the e-skin bonded.

To accommodate the elastic strains at the folded region, the Cu/PI traces within the e-skin are designed in a serpentine pattern, aligned with the longitudinal direction along the hinge axis (marked in Fig. 1b). This configuration enables the serpentine wires to undergo deformations in the longitudinal direction without compromising the integrity of the e-skin’s materials by reducing the wrinkling after stretching the traces. In addition, the serpentine wires are interconnected at several points using PI traces in the transverse direction. This interconnection strategy facilitates ease of handling and ensures the overall structural robustness of the e-skin, while reducing the overall weight of the e-skin compared to a fully connected PI layer.

A finite element analysis (FEA) was conducted to simulate the behavior of the structure under bending conditions. A comprehensive description of the simulation setup can be found in the method section. Figure 1d presents the resulting strain values in the longitudinal direction and transverse direction under both equal-sense and opposite-sense bending. It can be observed that the induced strain remains well below the elastic limit of copper (1%)^15^ and polyimide (3%)^16^. Consequently, the e-skin, composed of copper- and polyimide-based materials, is expected to avoid plastic deformation. The results can be further compared to FEA results of a bare tape-spring hinge without e-skin (Supplementary Fig. S2). Differences in the strain fields mainly exhibit in the areas of the patterned circuits. Compared to the bare tape-spring hinge, the strains in these locations increase by a negligible value of 0.24%. The overall strain distributions and maximum strain values are not changed after including the soft devices.

The active sensing system integrated into the e-skin consists of two microelectromechanical system (MEMS)-based accelerometers (Analog Devices, ADXL335) positioned at the two ends of the tape spring, along with a MEMS-based gyroscope (STMicroelectronics, LPR503AL) located at the tip. The circuit for the MEMS sensors is shown in Supplementary Fig. S1. Additionally, a set of three strain sensors is incorporated along the hinge longitudinal direction within the hinge region. These strain sensors utilize thin-film composites composed of reduced graphene oxide and single-walled carbon nanotubes (rGO/SWNT) as the sensing material for strain detection. The nominal mass of the 6-inch tape-spring segment is 3.6 ± 0.1 g, where the additional materials of e-skin have a nominal mass of 0.36 ± 0.02 g. It is also worth mentioning that components of the e-skin are not along the center line.

Recent research efforts have yielded various thin-film sensors based on rGO. For example, Ye et al. employed a self-assembly approach to fabricate a polyetherimide-rGO strain sensor and achieving a maximal gauge factor of approximately 754 at 5% applied strain^17^. Similarly, Wang et al. utilized a resistive-type thermoplastic polyurethane-rGO strain sensor for human motion monitoring^18^. In addition to its excellence in sensitivity, the stretchability of such material can also be tailored to accommodate large strains. Lou et al. developed a series of Brij-35-PDMS/rGO/CNT stretchable strain sensors that demonstrated a controllable sensing range from 55% to 210% applied strain^19^. Luo et al. developed stretchable rGO/CNT/TPU (thermoplastic polyurethane) sensors that achieved a stable linear sensitivity at 50% applied strain with a gauge factor of approximately 75, which survived and showed stable responses for more than 1000 stretching cycles^20^.

Additionally, several studies have reported the development of rGO/CNT-based thin films, often combined with metal oxides to achieve additional functionalities such as piezoelectric effects or gas sensing^21–23^. The expected strains of the folded tape spring hinge are between ±0.5% (maximum at the corners of the bent region) in this work. Therefore, rGO/carbon nanotube-based sensors’ advantage in achieving a high gauge factor will be beneficial to sensing such small strains. Therefore, we have chosen rGO/carbon nanotube composite as the active material for strain sensing in this work.

The strain sensor we investigated in this study is a rGO/SWNT thin-film resistive-type strain sensor. The fabrication of this sensor is highly customizable to be compatible with the fabrication of other materials that are adopted in this study. With a drop-casting method, we can extensively reduce the fabrication complexity and achieve a gauge factor of approximately 50. To fabricate the strain sensors, we mixed the rGO solution and the SWNT solution, which were then drop-casted onto a PDMS mold in the desired area. Following the deposition, the remaining solvent was evaporated, allowing the rGO/SWNT mixture to form a thin film on the substrate. The thickness of the resulting film can be controlled by adjusting the amount of liquid mixture employed in the mold. This fabrication method is highly feasible and user-friendly. Section “Fabrication of rGO/SWNT strain sensors” includes more details on the fabrication process.

### Monitoring quasi-static folding and deformation of tape-spring hinge

**Fig. 2:**
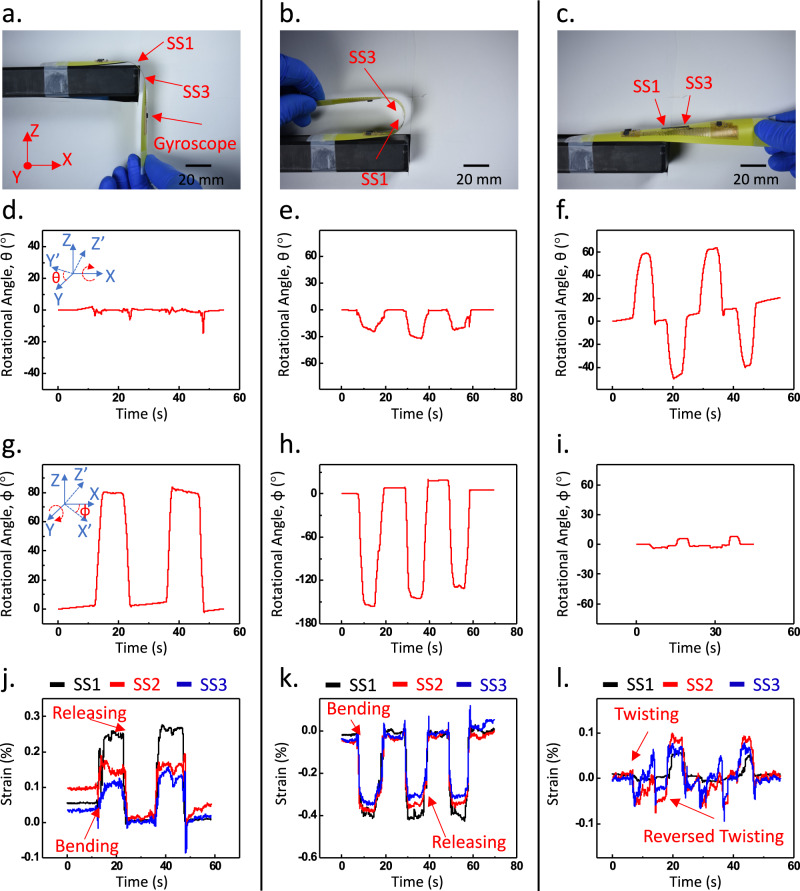
Monitoring quasi-static folding and deformation of tape-spring hinge. **a**–**c** Quasi-static bending and twisting of tape-spring hinge. (SS: Strain Sensor). **d**–**f** Rotation angle around the longitudinal direction of the hinge during bending and twisting. **g**–**i** Rotation angle around the transverse direction of the hinge during bending and twisting. **j**–**l** Longitudinal strains measured by the strain sensors during bending and twisting.

The e-skin is employed to monitor the quasi-static folding and deformation of the tape-spring hinge, as depicted in Fig. 2. The hinge undergoes slow manual folding and deformation in a cyclic, quasi-static manner, while the gyroscope and strain sensors capture the rotation of the hinge tip and longitudinal strains in the folded region, respectively. The deformations of the hinge result in the rotation of hinge tip, where the rotational velocity is converted into a voltage output by the gyroscope. By numerically integrating the measured angular acceleration, the rotational speed and angle of the hinge tip can be calculated.

Figure 2a, d, g illustrates the equal-sense folding process and present the measured rotation angles around the *x*- and *y*-axes. The *x*-axis data (Fig. 2d) represents the twisting motion along the longitudinal direction of the tape-spring hinge, while the *y*-axis data (Fig. 2g) represents the rotational angle around the transverse direction. As the equal-sense folding occurs, the hinge undergoes a clockwise rotation from a horizontal position to a nearly downward position while minimizing the twisting along the *x*-axis. While directly measuring the voltage output from the gyroscope, results in Fig. 2d–i were calculated by subtracting the nominal baseline voltage provided by the manufacturer from the measured voltage and then converted to angular speed with a linear relationship 8.3 mV dps^−1^. The resulting angular velocity was then integrated to obtain the cumulative rotational angle in both *x* and *y* axis. The presented data were smoothed with a 2000-points FFT filter with cutoff frequency of 2.5 Hz to reduce disruptions due to vibration of the tape-spring hinge during motion.

Figure 2b, e, h showcase the opposite-sense folding process and display the measured rotation angles around the *x*- and *y*-axes and. The tape-spring hinge is folded upwards from its original shape to a nearly horizontal position (Fig. 2b) while introducing a slight twisting motion around the *x*-axis to evaluate the effectiveness of the e-skin in measuring multiple degrees of freedom rotations. The results depicted in Fig. 2e, h demonstrate that the e-skin successfully captures the rotations of the hinge tip in both the x-direction (twisting) and y-direction (folding). Additionally, a cyclic twisting motion is introduced in Fig. 2c, and the e-skin accurately measures the rotations of the hinge tip in both the *x*- and *y*-directions associated with the twisting motion. Overall, these results highlight the capability of the e-skin as a soft functional layer for sensing the motion of the tape-spring hinge.

The longitudinal strains occurring in the folded regions are monitored by the three rGO/SWNT strain sensors integrated into the e-skin. These mechanical strains induce changes in the resistance of the rGO/SWNT sensors, which are then converted into voltage signals as the output. To establish the calibration between the sensor voltage readings and the corresponding strain values, the strain in the folded region is correlated with the voltage reading of a strain sensor located at the same position. The rGO/SWNT sensors demonstrate an average gauge factor of 50, indicating their sensitivity to strain measurements. A nominal strain sensor calibration curve is shown in Fig. S4, where the resistance change of the strain sensor is recorded at various strains.

During the equal-sensing folding process (Fig. 2a), strain sensor (SS) 1 is positioned within the folded region, while SS2 and SS3 are located farther away from the folded region. As anticipated, SS1 exhibits the highest strain readings, whereas SS3 shows the lowest strain values (Fig. 2j). Additionally, all strain sensors display a positive strain (tensile) values below 0.3%, indicating stretching in the longitudinal direction, which aligns with the results obtained from finite element analysis (FEA). Similarly, during the opposite-sense folding (Fig. 2k), the strain sensors detect longitudinal compressive strains (negative values). The maximum compressive strain measured is less than 0.4%, which is consistent with the FEA result in Fig. 1d. The strain developed in the hinge area is mainly due to the curvature change of the tape spring in that area. A folded tape spring can be separated into three sections: an undeformed area, a fully developed folding area, and transition areas in between^24^. In the fully developed area, the strain value will remain constant as the curvature of the tape spring in this area will not change during the folding^25^, thereby resulting in constant strains. As we fold the tape spring to a larger angle, the fully developed area will become longer, but its strain will not change. Therefore, we expect that, if the tape spring is extended to 180°, the strains will remain constant and below material limits. On the other hand, the twisting motion of the hinge induces significantly smaller strains due to minor changes in the curvature of the hinge (Fig. 2l).

To validate the measurements from the strain sensors, a Digital Image Correlation (DIC) measurement is performed on a bare tape-spring hinge. Supplementary Fig. S3 shows the experimental setup and the DIC results. The DIC results suggested that the tensile strain is 0.33% at sensor locations, whereas the sensors show 0.3% tensile strain in the measurement (~10% error), showing a good agreement. The difference in the experimental setups has a major contribution to the error. For the DIC setup, it is required that the hinge area should remain at the focal plane of the cameras and should be kept stationary for clear images. Therefore, to hold the bent tape-spring hinge on the support beneath it, higher forces are needed compared to freely bending the hinge without the support. This increases curvature and creates a slightly larger strain values in DIC measurements compared to the strain sensor readings.

Over the course of this study, the strain sensors maintained a stable performance on the order of 100 cycles. Previous study conducted by others also showed a type of stretchable rGO/CNT/TPU (thermoplastic polyurethane) sensors that survives and shows stable responses for more than 1000 stretching cycles^20^. Therefore, it is promising that the maximum cycles that the sensors can survive should exceed general mission requirements. However, the deployable hinges studied in this work generally require a single successful deployment for spacecraft applications rather than multiple cycles. Due to the nature of this specific application, we did not perform cyclic folding and deployment testing.

### Monitoring the sliding of folded hinge

**Fig. 3:**
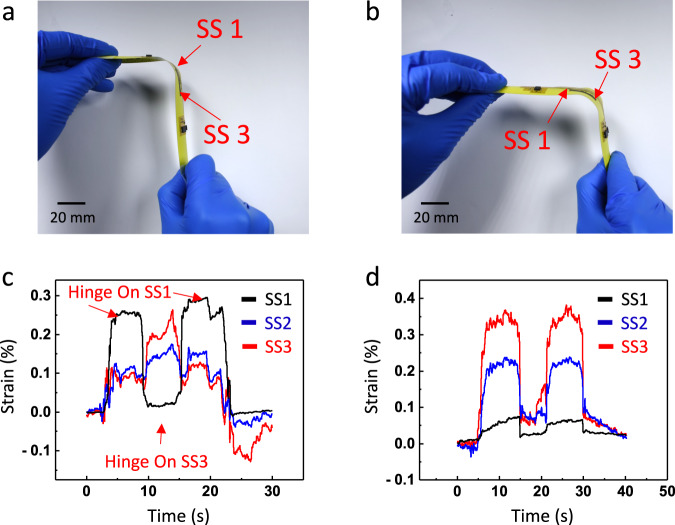
Monitoring the sliding of the folded hinge. **a** Equal-sense bending with folded region closer to strain sensor 1. **b** Equal-sense bending with folded region closer to strain sensor 3. **c** Strains during folded region sliding between strain sensors 1 and 3; **d** Strains of the folded region sliding around strain sensors 2 and 3. (SS: Strain Sensor).

One limitation of tape-spring hinges is the potential for the folded region to slide under the applied bending moments, resulting in uncertain locations of the folded region^26^. Hence, it is crucial to monitor the sliding of the folded region. As shown in Fig. 3a, b, the folded region exhibits movement while the orientations of the hinge tips remain unchanged. Consequently, the gyroscope attached to the e-skin on the hinge tips is unable to detect the sliding of the folded region. Furthermore, rigid MEMS-based devices like the gyroscope cannot be attached to the folded region due to its substantial deformation. In this study, we demonstrate that the three rGO/SWNT strain sensors can effectively serve as a sensing system for monitoring the sliding of the folded region.

Figure 3c shows the strains measured by the rGO/SWNT strain sensors during cyclic sliding of the folded region. Initially, when the folded region is positioned at SS1, a strain reading of 0.3% is observed, which is higher than the strains measured at SS2 and SS3 due to their distance from the folded region. As the folded region slides closer to SS2 and SS3, higher strains are recorded by these sensors compared to SS1. Note that the folded region was manually held for ~5 to 10 s at locations near SS1 and SS3, and the sliding motions in between were quick. Therefore, strain plateaus were clearly captured in all sensors at the static holding periods; however, we did not record enough data during sliding to clearly capture the responses of the sensors in these fast motions.

To show that once the hinge area is stabilized and not allowed to move along the tapespring, the sensor shows expected response, Fig. 3d includes cyclic strain measurements after the folded region moves away from SS1 and approaches SS3. The strains detected by SS1 remain below 0.05%, indicating that the folded region is no longer in proximity to SS1. The cyclic strains measured by SS2 and SS3 demonstrate that the folded region moves closer to their respective locations. Notably, SS3 reaches a maximum strain of 0.3%, while SS2 only reaches a strain of 0.2%, suggesting that SS2 is not entirely within the folded region. Therefore, based on the strain data shown in Fig. 3d, it can be inferred that the folded region slides near SS3, reaches SS2, but does not pass beyond SS3.

### Monitoring deployment dynamics

**Fig. 4:**
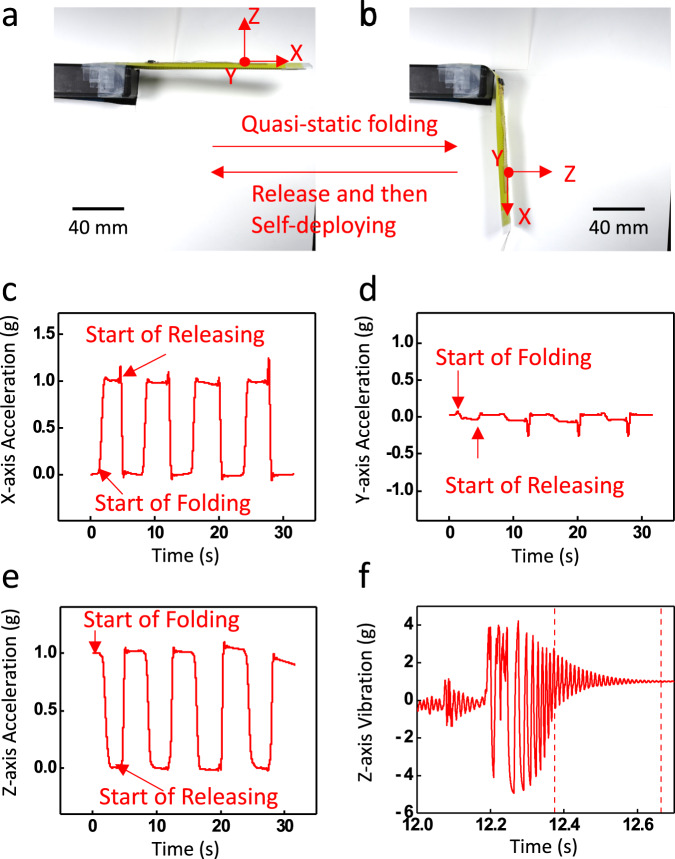
Monitoring of dynamics during folding and self-deployment using the accelerometer of the e-skin. **a**, **b** Images of experimental setup. **c**–**e** Motion acceleration in *x*-, *y*-, and *z*-directions measured by the accelerometer on the e-skin. **f** *Z*-axis acceleration voltage output immediately after the tape-spring hinge is released. The region between the dotted lines is selected for the frequency analysis in Fig. 5.

We use the e-skin to measure the fast self-deployment process of the hinge and analyze the vibrational modes and frequencies after the hinge is fully deployed. Figure 4a, b shows the experimental setup for this study. The hinge is first folded to 90° in equal-sense and then released to allow self-deployment. The MEMS-based accelerometer serves as the sensor for measuring the folding and deployment process. The process is repeated four times in this study. The detailed experimental setup can be found in Supplementary Fig. S5. The equipment and software in the experimental setup are included in Table S1. Note that the accelerometers are not on the center line of the tape-spring hinge but parallel to the longitudinal direction.

When the hinge is at a fully deployed state (Fig. 4a), the *z*-axis measurement shows a reading equivalent to 1.0 g, which corresponds to the gravitational acceleration. When the hinge is bent downward (Fig. 4b), the gravitational acceleration will shift from z-direction to x-direction. Therefore, the z- and x-direction data will show alternating acceleration between 0 and 1.0 g, as shown in Fig. 4c–e. Raw data collected by the accelerometer were subtracted by the baseline provided by the manufacturer and converted to acceleration in the unit of g through a linear relationship, 300 mV g^−1^. Note that the data presented in Fig. 4c–e are smoothed with a 2000-point FFT filter with a cutoff frequency at 2.5 Hz to eliminate the effect of the vibration of the tape-spring hinge to demonstrate the quasi-static behavior. To further analyze the dynamic response of the vibration of the tape-spring hinge. A zoomed-in section of the measured unsmoothed data from the accelerometer immediately after the dynamical release is shown in Fig. 4f. The amplitude of the acceleration due to the vibration of the tape-spring hinge is approximately within ±4 g, which is much greater than the gravitational acceleration. The oscillation of the *z*-axis voltage reading indicates a dynamic vibration of the structure, which diminishes after approximately 0.5 s.

**Fig. 5:**
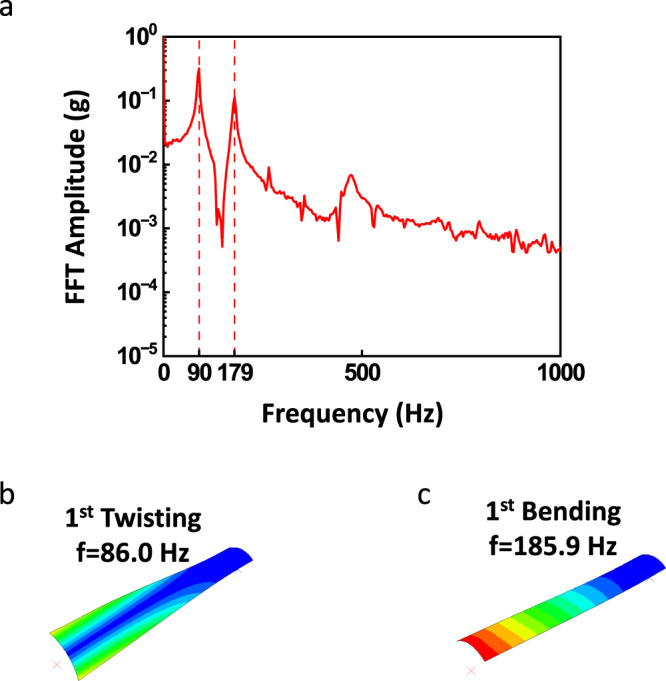
Modal analyses using experimental data collected from the e-skin and FEA. **a** FFT result of z-axis vibration. **b**, **c** FEA results showing the first vibrational modes at frequencies of 86.0 Hz and 185.9 Hz.

A fast Fourier transform (FFT) was performed on the vibration captured by the accelerometer in its *z*-axis (Fig. 5a). We obtained the first two vibration modes at 90 Hz and 179 Hz. The data captured in the *x*- and *y*-axes of the accelerometer have much lower amplitudes compared to the vibration in the *z*-direction. We performed a frequency analysis using Abaqus/Standard to compare to the measured modes. The simulated tape-spring hinge has the same geometry dimensions as the one mentioned in the mechanical analysis. A detailed simulation setup can be found in Section “Frequency Analysis”. The first two modes of vibration calculated by the FEA analysis are shown in Fig. 5b, c, with corresponding natural frequencies of 86.0 Hz and 185.9 Hz. The measured frequencies match well with the simulations with less than 5% error. The measured frequencies deviate from the FEA results because the FEA simulations cannot fully replicate the actual boundary conditions in the experiments. The simulated boundary condition was a perfect clamp at the root of the tape-spring hinge where no elastic deformation was allowed in the clamped region. However, the hinge was clamped with a PLA (polylactic acid) clamp during the experiment that allowed elastic deformation of the hinge at the root. Additionally, the tape-spring hinge is fixed on a shaker to reduce the damping of the quasi-static vibration, which provides additional vibration cycles for the FFT analysis, therefore, higher FFT resolution. Therefore, a system level design of the simulated boundary condition will be necessary to accurately represent the experimental conditions.

## Conclusion

In this study, we introduced a concept of structural e-skin for in-situ monitoring of the folding and dynamic behavior of self-deployable tape-spring hinges. Through the development of innovative soft electronics fabrication techniques, we successfully manufactured the e-skin, which comprised soft circuitry, high-gauge-factor thin-film rGO/SWNT strain sensors, and MEMS-based gyroscope and accelerometers. The experimental results, coupled with finite element analysis (FEA) simulations, showcased the e-skin’s ability to accommodate large deformations in folded hinge areas.

Our study demonstrated that the e-skin was capable of accurately capturing both the quasi-static folding and deployment motions and the strains in the folded hinge region. The strain sensors effectively detected the sliding of the folded hinge regions, providing valuable insights into the behavior of the structure. Additionally, the e-skin successfully captured the dynamic deployment behavior of the tape-spring hinge, accurately identifying the first two vibration modes of a 6-inch tape-spring hinge. These results were further validated through FEA frequency analysis.

The significance of this research lies in its proposal of a promising approach that leverages the design and manufacturing of soft electronics for highly deformable thin-walled deployable structures. By offering thin, lightweight, and soft functional devices with multifunctionality, the e-skin addresses the requirements of such structures for multifunctional devices to monitor their behavior. Furthermore, our work establishes a foundation for future research directions in the field of soft electronics for space structures.

Several potential avenues for future exploration emerge from this study. These include investigating the effects of space environments on soft electronic devices and developing strategies to protect them against adverse conditions. Our previous work has demonstrated that MEMS-based sensors can function at thermal-vacuum conditions that simulate low-Earth orbit (LEO) environments, and these sensors typically have integrated temperature sensors and self-compensation ability to accommodate temperature variations^27^. Resistive-type strain sensors may be affected by temperature variations and the thermal effects can be compensated by calibrating the changes in the baseline resistance due to temperature fluctuations.

Long-term operation in space is a challenge for thin-film sensors and many other space materials. Protective materials can be deposited to the sensor surface to increase the operational duration. For example, hydrogen-rich materials^28^, silicon dioxide (SiO_2_), and silicon nitride^29^ encapsulation can be used to protect the sensors from atomic oxygen and ultraviolet radiation. Additional polyimide encapsulations are also commonly used as sacrificial layers to increase the lifetime of essential functional materials. The thickness and compositions of these materials can be controlled through spin-coating and evaporation deposition to achieve composite layups from sub-micron to tens of micrometers, thereby offering a wide range of design domains to find the optimal encapsulation to meet the specific mission requirements. For rGO/SWNT strain sensors, encapsulating with polyimide and SiO_2_ can achieve more durable sensors in space. We do not expect that it will affect the sensitivity, but it will sacrifice the flexibility of the sensing material and add extra weight and thickness. In practical applications, durability must be carefully designed within the constraints of thickness, flexibility, and weight of these devices. Design, fabrication, and optimization of protective materials for these sensors are important research directions to explore in the future.

Secondly, structural shape reconstruction is also a challenging direction that needs more in-depth studies in the future. With a strain sensor array that spans the entire structure surface, it is possible to plot the strain distribution at the surface of the structure, therefore, relates to the deformation of the structure at every location. A high-density array of sensors will be necessary to achieve high-resolution strain sensing for shape reconstruction. Accelerometers and gyroscopes can provide additional information on the mechanical behavior of the structure. Shape reconstruction will inevitably be more challenging as the complexity of the targeted structure increases. In the specific applications that require shape reconstruction of highly deformable structures, the sensor network will need to be carefully designed considering the requirement of accuracy and limits on weight. The e-skin presented in this work may serve as the constituent nodes of such sensor networks that can be used to reconstruct shapes.

Additionally, future research could focus on embedding similar concepts within deployable composite structures, expanding the range of sensing and actuation modalities, and exploring various other innovative possibilities. The current e-skin design may be modified to achieve a longer distance of data and power transmission. Our previous work has demonstrated that, by embedding ultra-thin wires within an ultra-thin composite boom, we were able to provide power to and extract data from a flexible electronics patch attached to the tip of a 4-feet-long boom^27^. An important and more challenging future research direction is to scale the e-skin to a large sensory system with extensive sensing nodes to cover an expansive area for high-resolution sensing. The size of the e-skin device is limited to a few inches due to the constraints of microfabrication techniques. Larger soft sensor systems may be obtained by using printing techniques such as conductive ink printing and roll-to-roll printing. A potential issue with a large sensor system is the additional weight and lengthy wire connections. A possible approach to mitigate this problem is to utilize an array of sensors consisting of modularized soft sensing nodes. A subgroup of nodes may be connected to local thin-film power sources, such as thin-film batteries and photovoltaic solar cells, and soft wireless communication circuitries to eliminate lengthy wires. For space applications that require extreme constraints on allowable add-on mass and shifted frequency, the soft e-skin integrated on space structures will need to be carefully designed based on the requirements and constraints to find an optimal solution balancing between sensing capability and sensor weight. However, it should be noted that the technologies of soft electronics provide a more promising method to reduce weight and achieve conformal devices compared to traditional rigid electronics with heavy and bulky substrates. By harnessing the capabilities of these technologies, it may unlock new opportunities for enhancing the functionality, reliability, and efficiency of deployable space structures in future space missions.

## Methods

### Fabrication of soft e-skin

#### Sample preparation of Cu/PI substrate

The Cu/PI substrate (Dupont AR1820) was affixed to a glass slide coated with polydimethylsiloxane (PDMS) to facilitate easy handling. To create the PDMS coating, spin coating was performed at 1000 rpm for 30 s, followed by baking at 110 °C for 1 min to partially cure the PDMS. Subsequently, the PDMS-coated glass slide with the attached Cu/PI substrate was placed in a 70 °C oven for a minimum of 2 h to ensure complete PDMS curing. Prior to subsequent processing steps, the sample surface was carefully rinsed with acetone and isopropyl alcohol (IPA).

#### Etching of Cu/PI substrate to fabricate soft circuitry

The Cu/PI substrate obtained from the previous step underwent patterning using photolithography. Initially, a thin film of AZ 5214 was spin-coated onto the Cu/PI substrate at 3000 rpm for 30 s. The desired circuit traces were defined through UV exposure of 60 mJ and subsequently developed using a 1:4 ratio of AZ 400 K Developer. Next, the exposed areas of the underlying Cu film were etched using Copper Etchant Type 100 (Transene). After the successful removal of all undesired Cu traces, the sample surface was carefully cleaned with acetone and IPA.

To create a shadow mask for plasma etching the PI film, a 50 nm Aluminum thin film was deposited on the back side of the Cu/PI substrate using a Temescal Deposition tool. The Aluminum film mask was then patterned using photolithography (AZ 5214, 3000 rpm, 30 s; 1:4 AZ 400 K Developer). Conveniently, the AZ 400 K developer was capable of etching the Aluminum film while etching the photoresist. The resulting sample was subsequently etched using a Vision 320 Plasma Etching tool (45 sccm O2, 5 sccm SF6, 200 W, 200 mTorr, 2 h). After removing the Aluminum mask, the patterned Cu/PI circuit was detached from the glass slide. Finally, the required electronic components were soldered onto the flexible circuit using low-temperature solder paste (TS391LT, Chip Quick Inc.).

#### Fabrication of rGO/SWNT strain sensors

To prepare the rGO/SWNT dispersion, 3 mg P2-SWNT powder (Carbon Solution) and 9 mg graphene oxide powder (Carbon Solution) were dispersed in 100 ml DI water by probe sonication for 0.5 h. Hydroiodic acid was then added to the dispersion to reduce graphene oxide into reduced graphene oxide. After centrifugation, all liquid was removed to get rGO/SWNT powder, which was then dispersed in 100 ml DI water via probe sonication for 0.5 h. The resulting solution was further reduced by N2H2 in a water bath at 90 °C for 12 h. To remove undesired solvent and unreacted chemicals, the rGO/SWNT dispersion was washed by vacuum filtration method using DI water and then re-dispersed in 100 ml 1% sodium dodecyl solution by probe sonication for 1 h. The final solution was then cast directly on the fabricated electrodes. PDMS molds were used to contain any liquid within the designated area.

### Finite element analysis

#### FEA simulations

The FEA analysis was conducted using Abaqus Standard software. The simulated tape-spring hinge was created as a 3D shell extrusion with dimensions of 152.4 mm in length and 0.115 mm in thickness. The tape-spring’s radius of curvature in an undeformed state was 16.9 mm, spanning an arc of 86 degrees. The material assigned to the hinge was steel, characterized by a density of 7.85 × 10^3^ kg m^−3^, a Young’s modulus of 210 GPa, and a Poisson’s ratio of 0.3.

To incorporate the e-skin design, the generated part was treated as a composite material. The e-skin components, namely PI (with a Young’s modulus of 2.5 GPa and a Poisson’s ratio of 0.3) and Cu (with a Young’s modulus of 117 GPa and a Poisson’s ratio of 0.3), were assigned to their respective partition areas to simulate their attachment to the tape-spring structure.

To replicate the actual behavior, each end of the tape-spring hinge was subjected to a 45-degree rotation boundary condition in Fig. 1, resulting in a deflection of the structure to a 90-degree angle at the midpoint. In-plane stress and strain were calculated within the analysis and exported as output for further evaluation and analysis.

#### Frequency analysis

FEA analysis was performed using Abaqus Standard software. The simulated tape-spring had the same geometry dimensions as the one mentioned in the mechanical analysis. The e-skin was included in the simulation. This model was clamped at one end with a 12.7 mm (0.5 inch) clamped region. Instead of a static mechanical displacement step, a linear perturbation step was used to solve the first 10 natural frequencies of the simulated tape-spring. The Lanczos eigen solver was used.

### Experimental setup

#### Quasi-static experiments

The following setup was used to collect data presented in Figs. 2 and 3. The tape-spring hinge was fixed to a table through a 3D-printed PLA (polylactic acid) clamp that fits the curvature of the hinge. The electronics and sensors on the e-skin were connected to an intermediate PCB (printed circuit board) through flexible adhesive wires. A DC power supply was used to provide 3 V power to the MEMS chips and to a half-Wheatstone bridge that supports the strain sensors, respectively. Continuous and simultaneous voltage measurements were performed using a 16-channel DAQ system (PL3516 PowerLab 16/35, AD Instrument) across all output channels: 3-axis acceleration outputs from the accelerometer, 2-axis angular velocity outputs from the gyroscope, and three half-Wheatstone bridge output for individual strain sensors.

#### Dynamic response experiment

This setup was used to collect data presented in Fig. 4. The DAQ setup remains consistent with the previous section, whereas the tape-spring hinge is fixed on top of a shaker (Mini SmartShakerTM model K2007E01, Supplementary Fig. S5). In this study, the shaker is used to soften the boundary condition to reduce the damping of the vibration of the tape-spring hinge for a higher resolution of the FFT result.

### Supplementary information

Supplementary Information

## Data Availability

All data that support the conclusions are presented in the paper. All relevant data are available from the corresponding author upon reasonable request.

## References

[1] Arya, M. et al., *Large-Area Deployable Reflectarray Antenna for CubeSats*, in *AIAA Scitech**2019**Forum*.

[2] Ferraro, S. & S. Pellegrino, *Self-Deployable Joints for Ultra-Light Space Structures*, in *2018**AIAA Spacecraft Structures Conference*.

[3] Soykasap Ö (2007). Analysis of tape spring hinges. Int. J. Mech. Sci..

[4] Mallikarachchi, H. & Pellegrino, S. *Design and Validation of Thin-Walled Composite Deployable Booms with tape-Spring Hinges, in 52nd AIAA/ASME/ASCE/AHS/ASC Structures, Structural Dynamics and Materials Conference*.

[5] Mallikarachchi HMYC, Pellegrino S (2011). Quasi-static folding and deployment of ultrathin composite tape-spring hinges. J. Spacecraft Rockets.

[6] Ye H (2017). Optimal design of a three tape-spring hinge deployable space structure using an experimentally validated physics-based model. Struct. Multidiscip. Optim..

[7] Jorgensen, J. et al. *Dynamics of an Elastically Deployable Solar Array: Ground Test Model Validation, in 46th AIAA/ASME/ASCE/AHS/ASC Structures, Structural Dynamics and Materials Conference*.

[8] Rothberg SJ (2017). An international review of laser Doppler vibrometry: making light work of vibration measurement. Opt. Lasers Eng..

[9] Yu L, Pan B (2017). Single-camera high-speed stereo-digital image correlation for full-field vibration measurement. Mechan. Syst. Signal Process..

[10] Giraudeau A, Guo B, Pierron F (2006). Stiffness and damping identification from full field measurements on vibrating plates. Exp. Mechan..

[11] Chamberlain MK (2021). On-orbit flight testing of the Roll-Out Solar Array. Acta Astronautica.

[12] Chen T (2018). Experimental study on cross-sensitivity of temperature and vibration of embedded fiber Bragg grating sensors. Optoelectron. Lett..

[13] Chung HU (2020). Skin-interfaced biosensors for advanced wireless physiological monitoring in neonatal and pediatric intensive-care units. Nat. Med..

[14] Yu X (2019). Skin-integrated wireless haptic interfaces for virtual and augmented reality. Nature.

[15] Zhu J, Feng J, Guo Z (2014). Mechanical properties of commercial copper current-collector foils. RSC Adv..

[16] McKeen, L. W., *7 - Polyimides*, in *Film Properties of Plastics and Elastomers (Fourth Edition)*, (ed McKeen, L.W.) 147–185 (William Andrew Publishing, 2017).

[17] Ye X (2017). A wearable and highly sensitive strain sensor based on a polyethylenimine–rGO layered nanocomposite thin film. J. Mater. Chem. C.

[18] Wang Y (2018). Flexible electrically resistive-type strain sensors based on reduced graphene oxide-decorated electrospun polymer fibrous mats for human motion monitoring. Carbon.

[19] Lou C (2022). Highly stretchable and self-adhesive elastomers based on polymer chain rearrangement for high-performance strain sensors. ACS Omega.

[20] Luo W (2020). Stretchable strain sensors based on conductive coating cracks with improved interfacial adhesion by wet phase separation treatment. J. Coat. Technol. Res..

[21] Liu S (2015). High performance room temperature NO2 sensors based on reduced graphene oxide-multiwalled carbon nanotubes-tin oxide nanoparticles hybrids. Sens. Actuators B Chem..

[22] Ahmed A (2019). Preparation of PVDF-TrFE based electrospun nanofibers decorated with PEDOT-CNT/rGO composites for piezo-electric pressure sensor. J. Mater. Sci. Mater. Electron..

[23] Zhu M (2019). Piezoresistive strain sensor based on monolayer molybdenum disulfide continuous film deposited by chemical vapor deposition. J. Micromechan. Microeng..

[24] Picault, E. et al. *On the folding and deployment of tape springs: A large displacements and large rotations rod model with highly flexible thin-walled cross-sections*, in *53rd AIAA/ASME/ASCE/AHS/ASC Structures, Structural Dynamics and Materials Conference*.

[25] Seffen KA, Pellegrino S (1999). Deployment dynamics of tape springs. Proc. R. Soc. Lond A Math. Phys. Eng. Sci..

[26] Miura, K. & Pellegrino, S. *Forms and Concepts for Lightweight Structures* (Cambridge University Press, 2020).

[27] Yao, Y. et al. *A Multifunctional Bistable Ultrathin Composite Boom for In-Space Monitoring of Deployment Dynamics*, in *AIAA SCITECH 2023 Forum*.

[28] Zeitlin, C. et al. *Ground-based Testing of Radiation Shielding Materials*, in *42nd AIAA Aerospace Sciences Meeting and Exhibit*.

[29] Tennyson RC (1994). Protective coatings for spacecraft materials. Surf. Coat. Technol..

