# Supplementary material for: Soft electronic skin for self-deployable tape-spring hinges
Source: Commun Eng. 2024 Jan 25;3:16. doi: 10.1038/s44172-024-00163-x (PMC10955954; doi:10.1038/s44172-024-00163-x)
Supplement: Supplementary file 1 — Supplementary Information [file 44172_2024_163_MOESM1_ESM.pdf]

Supplementary Information for:

Soft electronic skin for self-deployable tape-spring hinges

Yao Yao and Xin Ning

Department of Aerospace Engineering,
University of Illinois at Urbana-Champaign, Urbana, IL 61801

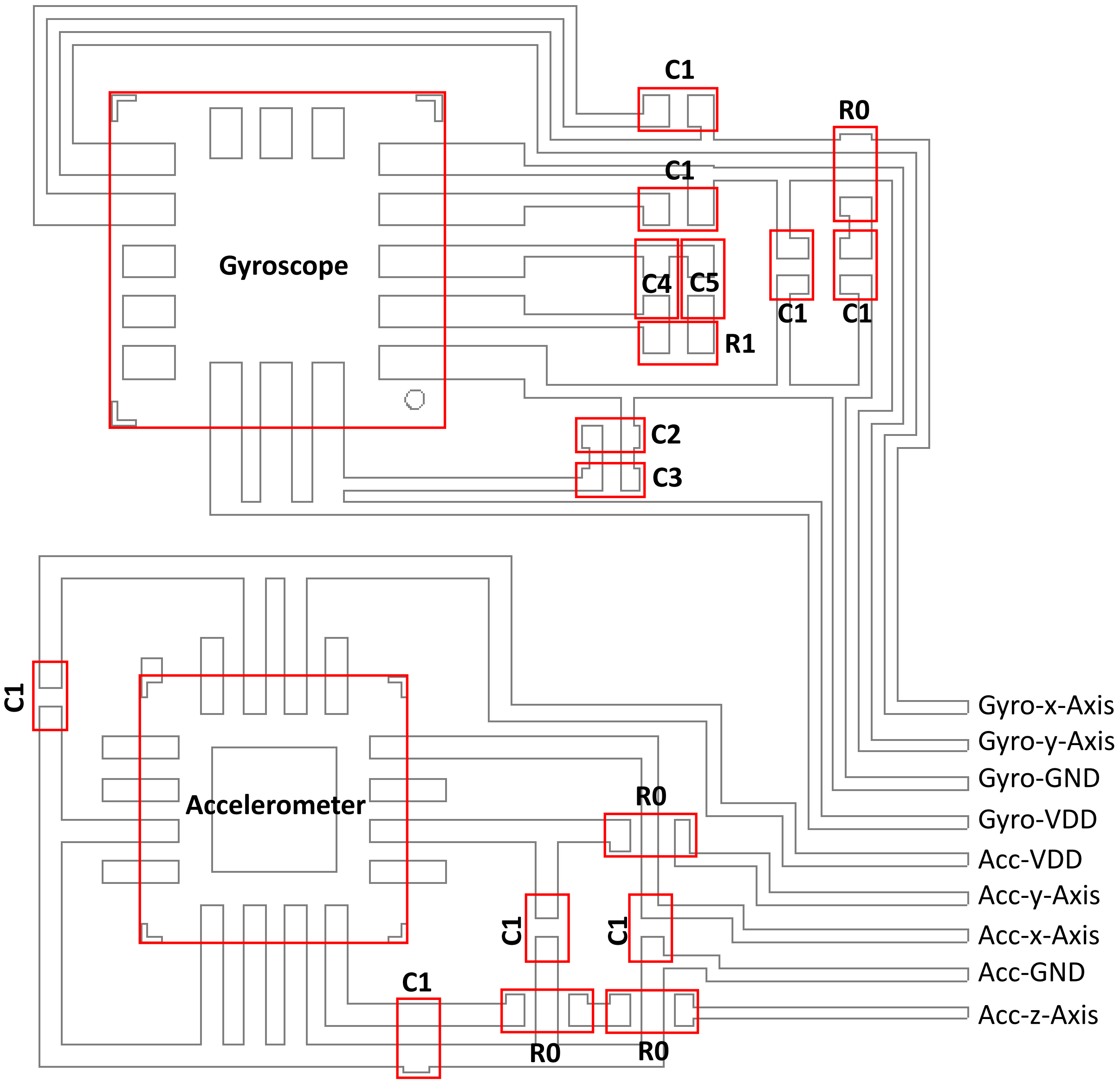

Figure S1. Connections of all electronic components used for gyroscope and accelerometer.

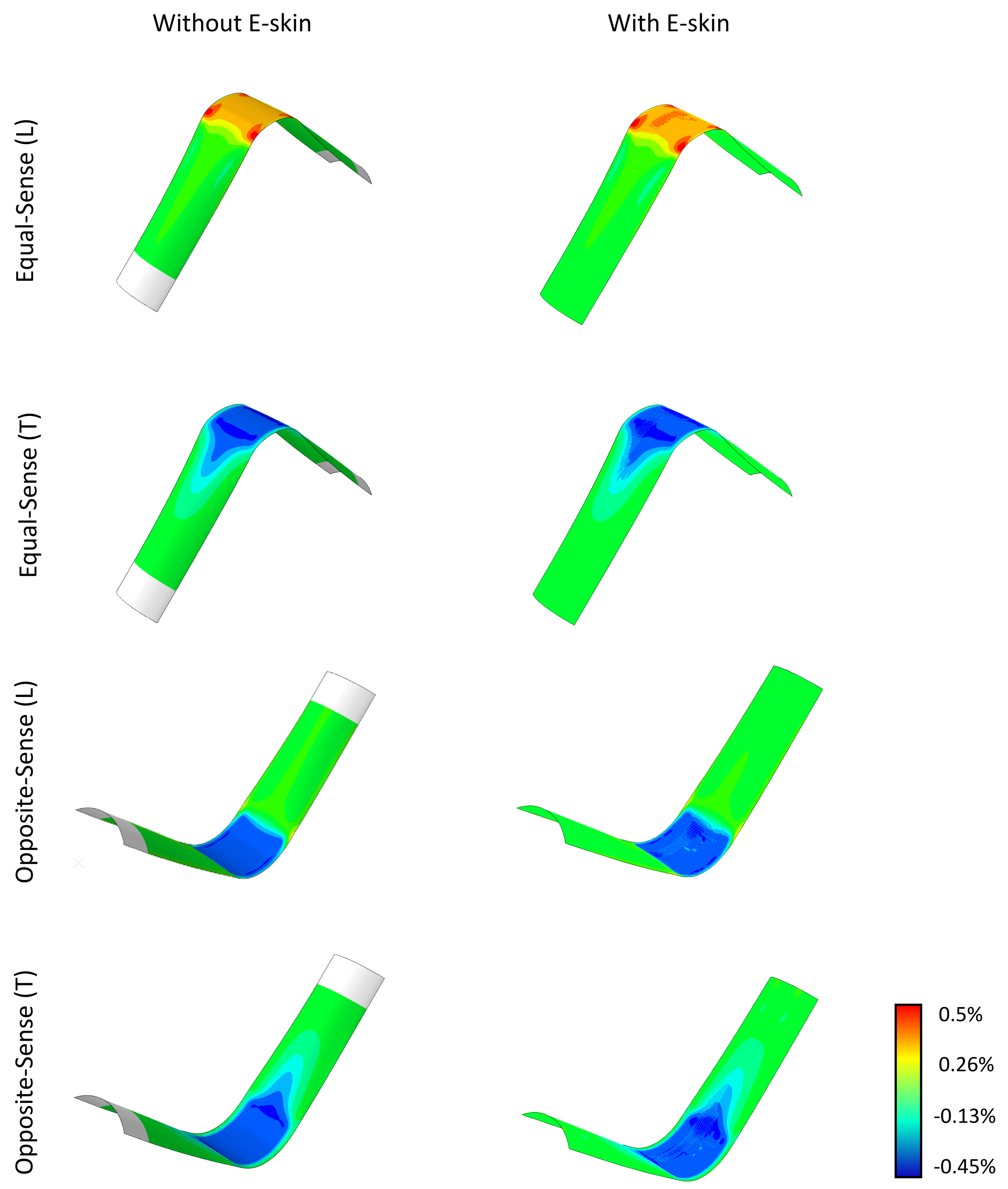

Figure S2. FEA result for longitudinal (L) and transverse (T) strains of both equal-sense and opposite-sense bending of bare tape-spring hinge without e-skin attached (left), compared to tape-spring hinge with e-skin attached (right).

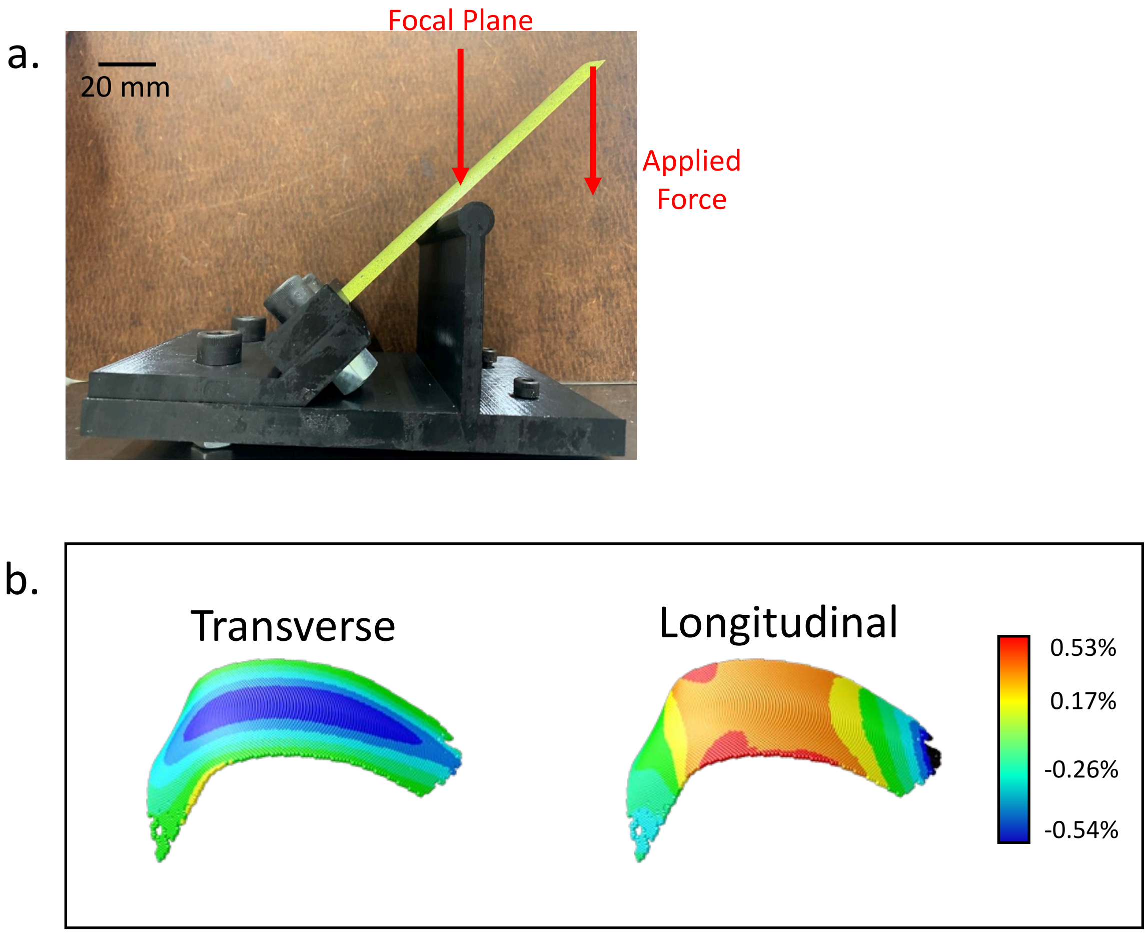

Figure S3. Digital Image Correlation (DIC) results of a tape-spring hinge under equal-sense bending. a) Experimental setup. The focal plane of the DIC cameras is set around the center of the hinge area. b) Strain measured through DIC on the bent tape-spring hinge surface.

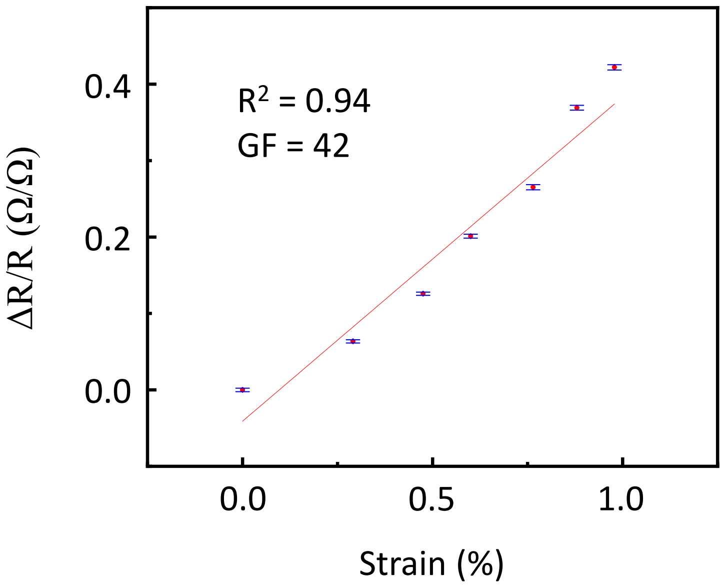

Figure S4. Nominal strain sensor calibration curve with linear fit (R^2=0.94, GF = 42).

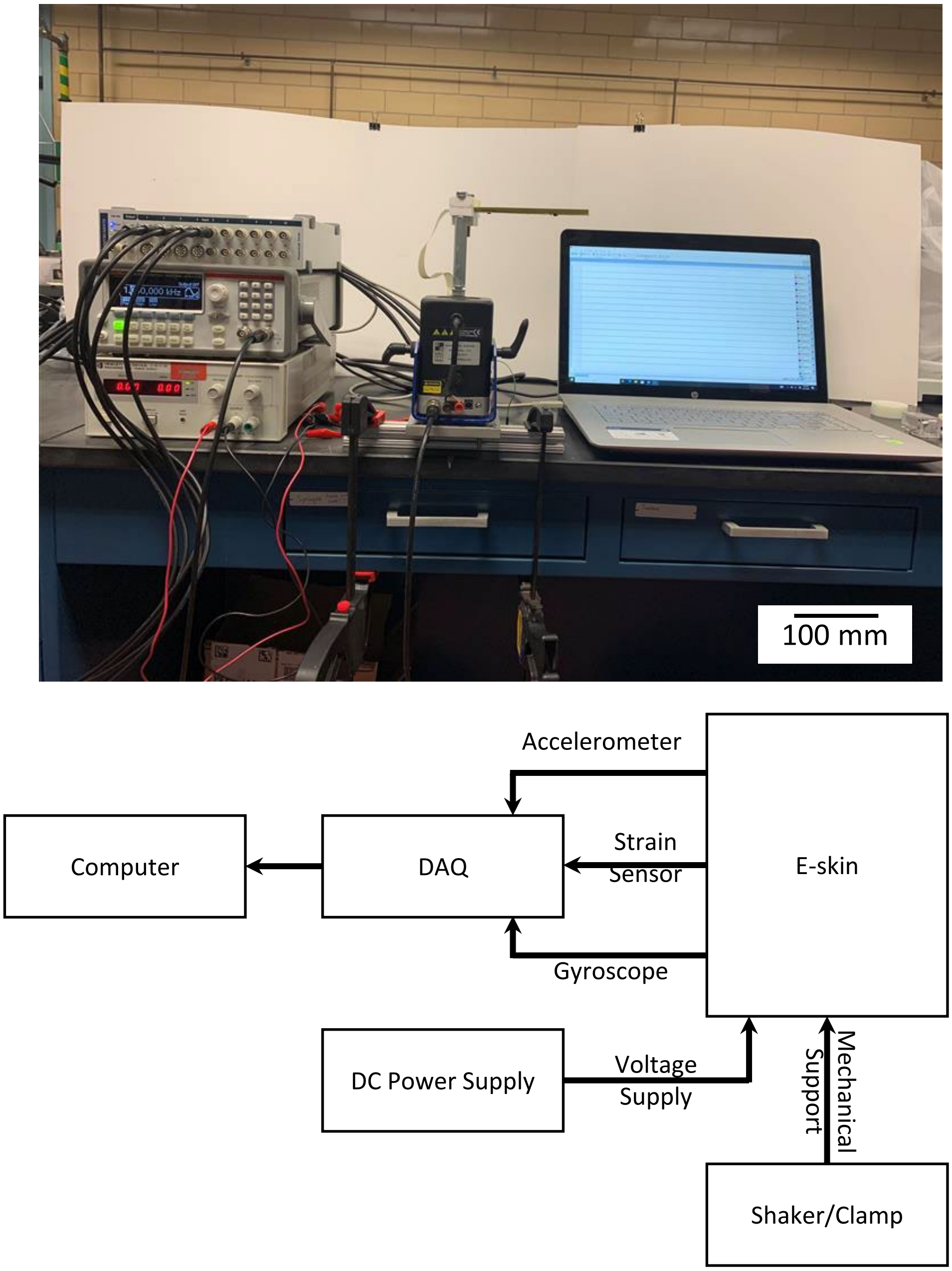

Figure S5. Image and diagram of experimental setup of vibration test.

Table S1. Lists of equipment and software used.

	Function	Model
**Equipment**	Analog to Digital Converter (DAQ)	PowerLab 16/35
	Shaker	Mini SmartShaker K2004E01
	DC Power Supply	Keysight E3630A
**Software**	Data Recording Through DAQ	LabChart 7
